# Microbial communities and arsenic biogeochemistry at the outflow of an alkaline sulfide-rich hot spring

**DOI:** 10.1038/srep25262

**Published:** 2016-04-29

**Authors:** Zhou Jiang, Ping Li, Joy D. Van Nostrand, Ping Zhang, Jizhong Zhou, Yanhong Wang, Xinyue Dai, Rui Zhang, Dawei Jiang, Yanxin Wang

**Affiliations:** 1 State Key Laboratory of Biogeology and Environmental Geology, China University of Geosciences, Wuhan, 430074, China; 2 School of Environmental Studies, China University of Geosciences, Wuhan, 430074, China; 3 Institute for Environmental Genomics and Department of Microbiology and Plant Biology, University of Oklahoma, Norman, OK, 73019, USA; 4 Earth Sciences Division, Lawrence Berkeley National Laboratory, Berkeley, CA, 94720, USA; 5 School of Environment, Tsinghua University, Beijing, 100084, China

## Abstract

Alkaline sulfide-rich hot springs provide a unique environment for microbial community and arsenic (As) biogeochemistry. In this study, a representative alkaline sulfide-rich hot spring, Zimeiquan in the Tengchong geothermal area, was chosen to study arsenic geochemistry and microbial community using Illumina MiSeq sequencing. Over 0.26 million 16S rRNA sequence reads were obtained from 5-paired parallel water and sediment samples along the hot spring’s outflow channel. High ratios of As(V)/As_Sum_ (total combined arsenate and arsenite concentrations) (0.59–0.78), coupled with high sulfide (up to 5.87 mg/L), were present in the hot spring’s pools, which suggested As(III) oxidation occurred. Along the outflow channel, As_Sum_ increased from 5.45 to 13.86 μmol/L, and the combined sulfide and sulfate concentrations increased from 292.02 to 364.28 μmol/L. These increases were primarily attributed to thioarsenic transformation. Temperature, sulfide, As and dissolved oxygen significantly shaped the microbial communities between not only the pools and downstream samples, but also water and sediment samples. Results implied that the upstream *Thermocrinis* was responsible for the transformation of thioarsenic to As(III) and the downstream *Thermus* contributed to derived As(III) oxidation. This study improves our understanding of microbially-mediated As transformation in alkaline sulfide-rich hot springs.

Hot springs provide a unique environment for examining the evolution of microbial communities in response to geochemistry as these environments harbor versatile microorganisms involved in various metabolic processes for hydrogen gas (H_2_), sulfur (S), iron (Fe) and arsenic (As), as well as having high temperature and a wide range of pH values^1^,^2^,^3^,^4^,^5^. Arsenic biogeochemistry has been studied extensively in acidic hot springs and it is well-documented that high concentrations of sulfide can inhibit microbial As(III) oxidation in acidic springs by inactivating expressed As(III) oxidase (AioA) in microorganisms such as *Hydrogenobacter*^6^, *Hydrogenobaculum*^7^ and *Acidicaldus*^8^. In contrast, sulfide has been shown to enhance microbial As(III) oxidation in the alkaline Mono Lake water by stimulating growth of sulfur-oxidizing bacteria, the first demonstration of the different roles of sulfide on microbial As(III) oxidation in acidic and alkaline environments^9^. Previous studies have revealed the presence of thioarsenic in alkaline sulfidic geothermal waters, and As and S oxidations were closely correlated in this type of system^10^,^11^. Thioarsenate was identified as the dominant As species at the source of the alkaline sulfide-rich hot springs in Yellowstone National Park (YNP) and account for up to 89% of total As^10^,^11^. Upon discharge, thioarsenate is biologically transformed to As(III) along the outflow channels, and is followed by appearance of As(V) and sulfate as the final products^12^,^13^. Studies of microbially controlled As transformation in mats or sediments in these alkaline sulfide-rich hot springs have been investigated using enrichments, pure cultures, clone libraries and metagenome sequencing and found *Thermocrinis*, *Thermus* and *Ectothiorhodospira* to be likely involved in transformation of As species^14^,^15^,^16^,^17^. As an example, anoxic cultures from alkaline Mono Lake and Big Soda Lake, which were dominated by *Ectothiorhodospira*, were able to use monothioarsenate as the sole electron donor for anoxygenic photosynthesis^16^. Further, the hyperthermophilic *Thermocrinis rubber* isolated from alkaline Octopus Spring in Yellowstone National Park (YNP) was shown to utilize monothioarsenate to aerobically grow, with the final products being arsenate and sulfate^17^.

Previous studies found that the microbial population in the water and sediment of hot springs varied depending on the specific geochemistry of the site, even within the same pool^18^,^19^,^20^. Although As biogeochemistry has been studied in hot springs as described above, arsenic geochemistry and the corresponding microbial communities in these alkaline sulfide-rich hot springs is still poorly understood. For example, the relationship between water and sediment geochemistry and the extent microbial communities is still not clear, with the As biogeochemistry and related genes remaining unspecified. To address these unknowns, the current study examined a representative alkaline sulfide- and As-rich hot spring located in the Rehai geothermal field of Tengchong, China^21^,^22^, and (1) systematically investigated the As geochemistry and microbial community structure in both water and sediments along the alkaline sulfide-rich hot spring outflow channel using Illumina MiSeq sequencing and clone libraries; (2) assessed the environmental factors shaping the microbial community structures; and (3) evaluated the potential microbially-mediated As transformation processes in the alkaline sulfide-rich hot spring.

## Results

### Water and sediment geochemistry

**Figure 1:**
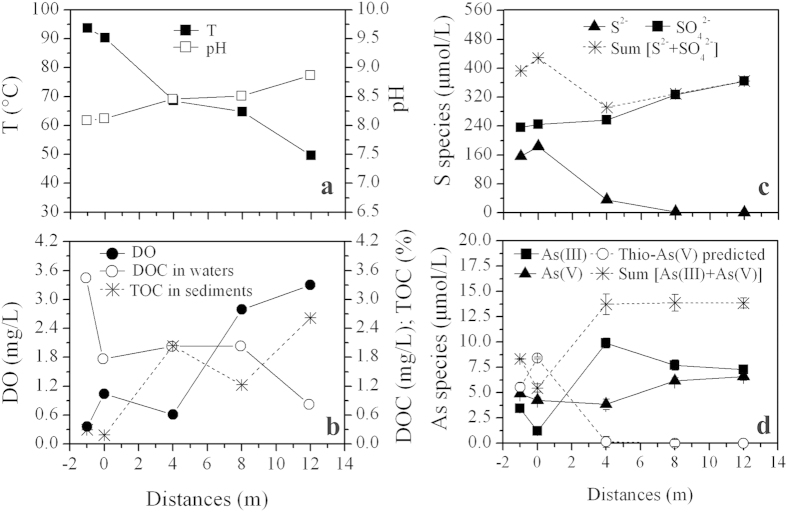
Distribution of selected geochemical data in water and sediment samples along the outflow channel of Zimeiquan. (**a**) T and pH; (**b**) DO and DOC in water samples and TOC in sediment samples; (**c**) sulfide and sulfate; (**d**) As(III), As(III), As_Sum_ and predicted thioarsenic. Error bars in Fig. 1d represent the standard deviations of duplicates.

Characterized by high sulfide concentrations of 156.25–183.44 μmol/L (or 5.00–5.87 mg/L), the two adjacent pools of Zimeiquan had similar pH (8.09–8.12), temperature (90.3–93.6 °C) and ions concentrations (Table 1 and Supplementary Fig. S1). A higher dissolved oxygen (DO) concentration and As(V)/As_Sum_ (the ratio between As(V) and the sum of As(III) and As(V)) were measured in the right pool (site 0 m) compared to left pool (site −1 m) (Table 1). Thioarsenic was detected in the pool water, with the major species being monothioarsenate (H_3_AsSO_3_), dithioarsenate (H_3_AsS_2_O_2_), and a low proportion of tetrathioarsenate (H_3_AsS_4_). Once the source water discharged into the outflow channel, most of the physicochemical parameters changed dramatically (Fig. 1). Temperature ranged from 93.6 °C to 49.6 °C along the outlet channel. The pH values slightly increased from 8.09 at −1 m to 8.87 at 12 m, which was primarily due to successive degassing (e.g. CO_2_ and H_2_S)^23^ and evaporation condensation^24^. DO, sulfate and As_Sum_ concentrations increased from 0.36 to 3.30 mg/L, 236.14 to 363.97 μmol/L and 5.45 to 13.86 μmol/L (or 0.62 to 1.11 mg/L), respectively. Dissolved organic carbon (DOC), ammonia and sulfide concentrations declined from 3.43 to 0.80 mg/L, 10.00 to 0 μmol/L and 156.25 to 0.31 μmol/L, respectively. As(III) concentrations increased from 1.20 μmol/L at 0 m to 9.89 μmol/L at 4 m and then gradually declined to 7.26 μmol/L from 4 m to 12 m. As(V) concentrations were practically unchanged from 0 m to 4 m, but increased from 3.84 to 6.59 μmol/L from 4 m to 12 m as did As(V)/As_Sum_ (0.28–0.48). Sediment Fe_Tot_ and As_Tot_ concentrations ranged from 29.39 mg/kg to 970.75 mg/kg and 22.15 mg/kg to 87.97 mg/kg, respectively (Table 1). Total organic carbon (TOC) concentrations increased (1.21–2.60%) in the downstream sediment samples.

### Alpha diversity of microbial communities

**Figure 2:**
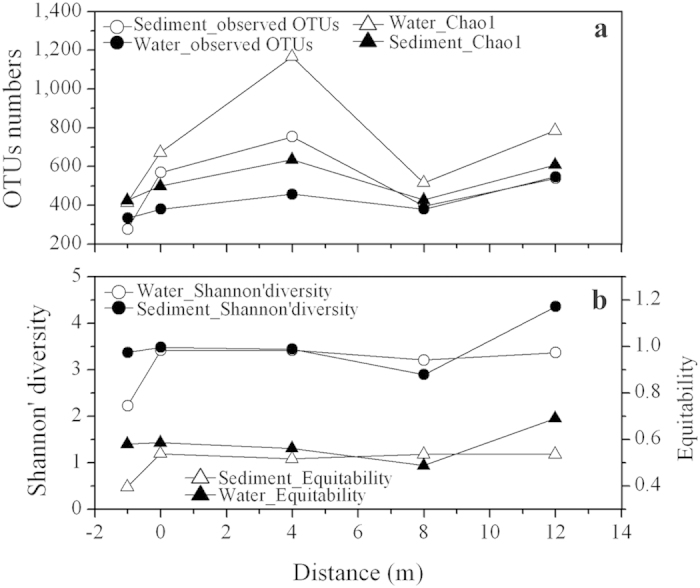
Alpha diversity indices distribution of microbial community structures of parallel samples collected along Zimeiquan outflow channel. (**a**) Observed OTUs numbers and Chao1. (**b**) Shannon’ diversity and Equitability. The solid and open symbols refer to sediment and water samples respectively.

A total of 256 883 sequences were initially obtained from the five-paired parallel water and sediment samples. After rarefaction at 15 000 sequences per sample, OTU clustering and removal of singletons, 148 019 sequences remained. A variety of taxa were present, with 277–754 observed and 414–1167 predicted OTUs (based on Chao1) and coverage values ranging from 64.63% to 89.96% (Supplementary Table S1). Water samples had higher microbial community richness than sediment samples, whereas Shannon diversity and equitability were relatively lower, except for site 8 m (Fig. 2). It should be noted that though a distinct increase of community richness was observed from −1 m to 4 m, Shannon diversity and equitability of all samples did not generally present a significant change along the outflow channel. Additionally, no significant correlation could be found between diversity indices and environmental factors (data not shown).

### Microbial community compositions and statistical analysis

**Figure 3:**
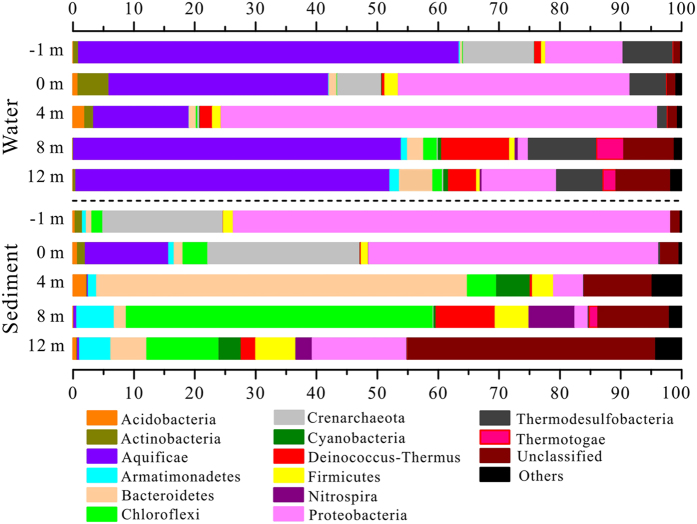
Microbial community structure of water and sediment samples along the Zimeiquan outflow channel at phylum level. Phyla with proportions higher than 4.5% are shown. Others named in the bar graph included phyla of *Verrucomicrobia*, *Caldiserica*, *Chlamydiae*, OD1, OP11, SR1, TM7, *Chlorobi*, *Dictyoglomi*, *Euryarchaeota*, *Fibrobacteres*, *Gemmatimonadetes*, *Planctomycetes*, and *Spirochaetes*.

Microbial community composition of the samples were distinctly different based on sample type (water vs. sediment) and by location (pools and downstream samples along the outflow channel) (Fig. 3). Water samples from the pools (−1 m and 0 m) were dominated by *Aquificae* (36.07–62.53%), *Proteobacteria* (12.73–38.07%), *Crenarchaeota* (7.21–11.68%) and *Thermodesulfobacteria* (5.97–8.24%). The parallel sediment samples also harbored the above phyla, but with significant different proportions: 47.67–71.89% *Proteobacteria*, 19.78–25.01% *Crenarchaeota* and 0–13.71% *Aquificae*. After discharge along the outlet, downstream water samples (site 4 m, 8 m, and 12 m) harbored *Aquificae* (15.69–53.87%), *Proteobacteria* (1.66–71.68%), and *Thermodesulfobacteria* (1.60–11.21%) as in the pool samples, but also diverse *Deinococcus-Thermus* (1.99–11.19%), *Bacteroidetes* (1.13–5.51%), *Thermotogae* (0.12–4.46%), and *Chloroflexi* (0.27–2.24%). Downstream sediment samples were mainly dominated by *Bacteroidetes* (1.96–60.90%), *Chloroflexi* (4.79–50.44%), and *Proteobacteria* (2.16–15.55%), with the remainder primarily composed of *Armatimonadetes* (1.38–6.16%), *Cyanobacteria* (0.37–5.50%), *Deinococcus-Thermus* (0.38–9.70%) and *Firmicutes* (3.47–6.60%). There were more unclassified phyla in the downstream sediment samples (11.19–40.71%) than in the corresponding water samples (1.53–8.99%).

**Figure 4:**
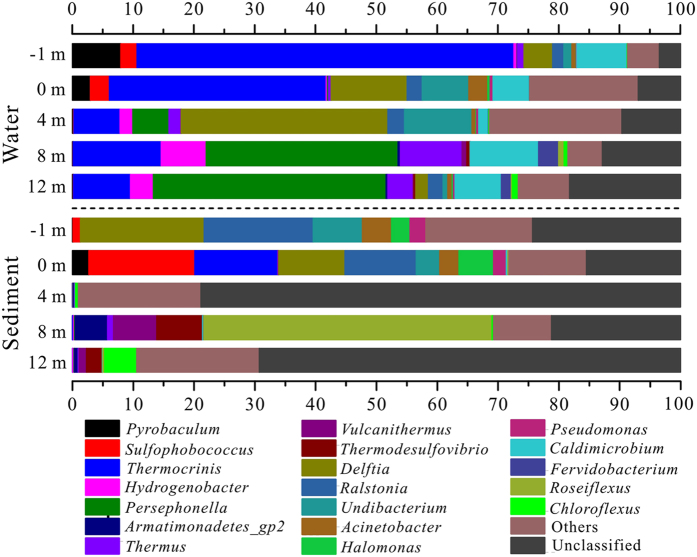
Microbial community structures of water and sediment samples along the Zimeiquan outflow channel at genus level. The ratios which exceeded 0.5% are displayed in the figure.

At the genus level, samples from pools (site −1 m and 0 m) were mainly comprised of *Thermocrinis* (13.68–61.97%) of *Aquificae*, *Ralstonia* (1.87–17.96%), *Delftia* (4.70–20.31%), *Undibacterium* (1.32–8.10%) and *Acinetobacter* (0.68–4.79%) of *Proteobacteria*, *Sulfophobococcus* (1.25–17.42%) and *Pyrobaculum* (0.08–7.96%) of *Crenarchaeota*, and *Caldimicrobium* (5.97–8.24%) of *Thermodesulfobacteria* (Fig. 4). Downstream water samples were mainly dominated by *Persephonella* (5.96–38.27%), *Thermocrinis* (7.57–14.48%) and *Hydrogenobacter* (2.11–7.41%) of *Aquificae*, *Thermus* (1.97–10.15%) of *Deinococcus-Thermus* and *Caldimicrobium* (1.58–11.20%) of *Thermodesulfobacteria*. Comparatively, most of the sediment derived sequences at 4 m and 12 m could not be assigned to known genus (69.27–78.87%). Only *Roseiflexus* (47.41%) of *Chloroflexi*, *Thermodesulfovibrio* (7.54%) of *Nitrospira* and *Vulcanithermus* (7.06%) of *Deinococcus-Thermus* were the primary genera detected in the sediment sample at 8 m.

**Figure 5:**
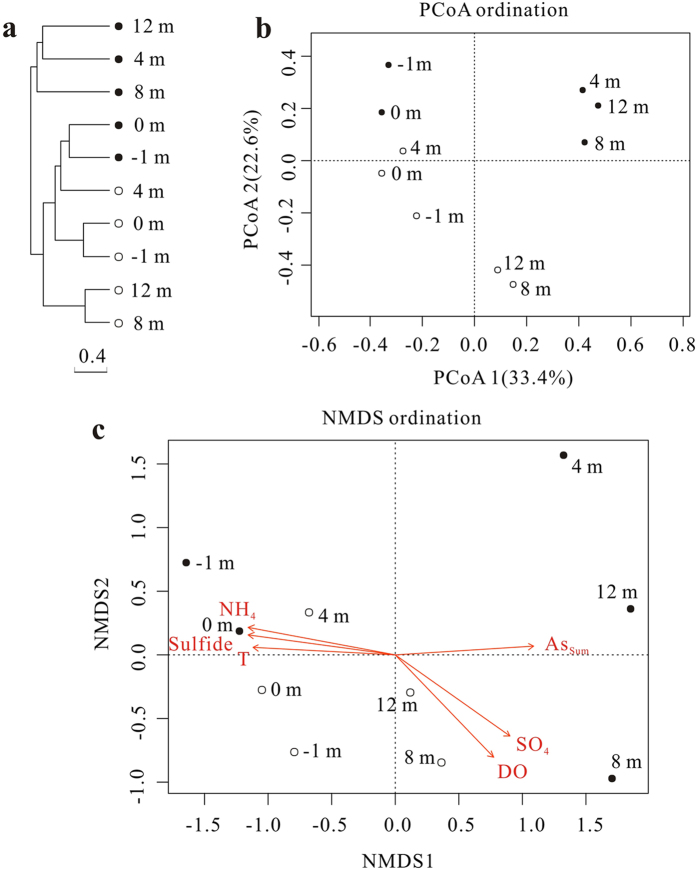
Microbial community distribution patterns at a 97% similarity level. All analyses were performed based on Bray-Curtis dissimilarity of normalized OTU abundances of samples. The solid circles and open circles represented sediment and water samples respectively. (**a**) The hierarchical cluster tree using the unweighted pair group method with arithmetic means (UPGMA). (**b**) Principal coordinates analysis (PCoA) scatter plot. The first two factors, PCoA1 and PCoA2, explained 33.4% and 22.6% of the observed variation. (**c**) Non-metric multidimensional scaling (NMDS) ordination plot. A biplot was overlaid on the ordination to identify environmental factors that were correlated with microbial community structure. The length of the line corresponds to the degree of correlation. Only variables that had a significant correlation (P < 0.05) are depicted.

Based on Bray-Curtis dissimilarity at a 97% similarity level, the UPGMA cluster tree of the microbial community populations showed that sediment samples were distinctly separated from the water samples and were separated into two groups (pools and downstream) (Fig. 5a). Similar results were also observed with the PCoA analysis based on the Bray-Curtis dissimilarity matrix and explained 56% of the observed variation (Fig. 5b). Consistent with these results, three complimentary non-parametric multivariate statistical tests including adonis, ANOISM, and MRPP revealed significant differences in microbial community structures not only between pools and the corresponding downstream samples, but also between water and sediment samples (Supplementary Table S2). Results of the Envfit function indicated that six geochemical parameters were significantly correlated (P < 0.05) with microbial community structure of the outlet samples, including temperature, DO, ammonia, sulfide, sulfate, and As_Sum_ with R^2^ values of 0.61, 0.61, 0.67, 0.66, 0.60 and 0.58, respectively (Fig. 5c). The similar direction of the ammonia, sulfide and temperature vectors, and the contrasting direction of the DO and sulfate vectors indicated correlations among these variables but did not necessarily suggest that all of the above environmental factors were responsible for the shift in the community structure.

### As(III) oxidization and As(III) oxidase gene (*aioA*) diversity

**Figure 6:**
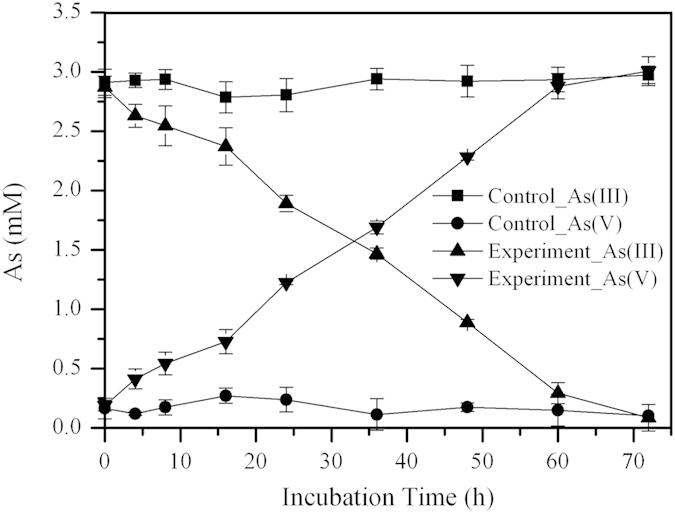
As(III) oxidation by enrichment product from Zimeiquan with minimal salt medium (MSM) containing 3 mM As(III). Experiments were carried out at 65 °C under chemolithoautotrophic conditions. Abiotic controls were mock inoculated with sterile water. Error bars are the standard deviation of triplicate values.

Consistent with the observed As(III) oxidation downstream, enrichment products from the downstream sample at 8 m was found to exhibit a strong capacity for As(III) oxidization under chemolithoautotrophic conditions. The enrichment products completely oxidized 3 mM As(III) in 72 h at 65 °C (Fig. 6). A total of 26 *aioA* gene clone sequences from this enrichment products displayed a high similarity (99%) and accordingly only one OTU was present in this library at a cutoff of 0.01. All encoded *aioA* were closely related (>90% amino acid level) to that of *Thermus* in the phylum *Deinococcus-Thermus* (Supplementary Fig. S2).

## Discussion

Sulfide can enhance microbial As(III) oxidation by stimulating the growth of indigenous sulfur-oxidizers in alkaline Mono Lake water^9^. The Zimeiquan pools are alkaline, sulfidic-rich, with As(V) as a dominant species. The high As(V)/As_Sum_ observed at this site suggests that As(III) oxidation occurred in these pools. This is a significant difference from previously studied acidic hot springs where microbial As(III) oxidation is potently inhibited by high sulfide concentration^8^,^25^, and highlights the distinct role of sulfide in As(III) oxidation in acidic and alkaline hot springs.

As_Sum_ significantly increased along the outflow channel. This might be due to evaporation condensation, dissolution from minerals, or transformation from thioarsenic. The lack of distinct change in representative ion concentrations (e.g., F, Cl, K and Na) along the outlet (Supplementary Fig. S1), and the slight fluctuation of As concentrations between sediments (Table 1) implies that As accumulation from evaporation condensation and mineral dissolution is negligible. Increased As(III) concentrations from 0 m to 4 m and subsequent decreases in concentration with a corresponding increase in As(V) from oxidation after 4 m (Fig. 1) suggest that the thioarsenate at this site is first converted to As(III) and then oxidized to As(V) after the thioarsenate disappears, as observed in previous studies^12^,^13^,^14^,^16^,^26^. Further, the reduced sulfur generated from the thioarsenate transformation was oxidized to sulfate as DO increased after 4 m, which led to the increase of S_sum_ from 4 m to 12 m (Fig. 1)^16^. Based on the As equilibrium observed in the three downstream sampling sites and the detection of monothioarsenate (H_3_AsSO_3_), dithioarsenate (H_3_AsS_2_O_2_) and tetrathioarsenate (H_3_AsS_4_) in the pools, we calculated that thioarsenate concentrations in the pools should be 5.5–8.4 μmol/L (Fig. 1d), and accounted for 39.9–60.7% of the total As. This predicted proportion of thioarsenate is consistent with the previously observed range of 31.2–89.0% in YNP^14^. These results implied that the increased As along the outflow channel is transformed from thioarsenate to As(III) and then oxidized to As(V).

**Figure 7:**
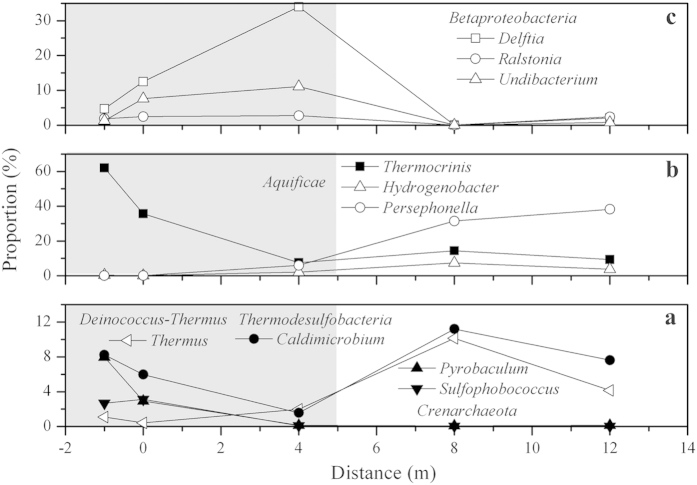
Distribution of dominant genera in water samples along the Zimeiquan outflow channel. (**a**) *Pyrobaculum* and *Sulfophobococcus* of *Crenarchaeota*, *Thermus* of *Deinococcus-Thermus* and *Caldimicrobium* of *Thermodesulfobacteria*; (**b**) *Thermocrinis, Hydrogenobacter* and *Persephonella* of *Aquificae*; (**c**) *Delftia, Ralstonia* and *Undibacterium* of *Betaproteobacteria*. Upstream sampling sites with relatively low DO concentrations (0.36–1.04 mg/L) are displayed in shadow.

Sequences with high similarity to the genus *Thermocrinis*, which are capable of mediating thioarsenic transformation, were dominant in our samples from −1 m to 4 m (Fig. 7) and played a role in transformaing of the thioarsenate to As(III) along the outflow channel. Previous studies showed that the transformation from thioarsenate to As(III) observed in alkaline sulfidic-rich Conch and Ojo Caliente springs in YNP were mediated by *Thermocrinis*-dominant microbial mats^13^,^14^. Furthermore, *Thermocrinis ruber* OC 14/7/2 isolated from Octopus Spring was found to be capable of using monothioarsenate as a sole electron donor for growth and then converting it to As(III) and As(V)^17^. In contrast, the As(III) oxidation observed downstream was attributed to the significant appearance of *Thermus* (Fig. 7). Evidence of microbially-mediated As(III) oxidation downstream was found with the enrichments and can be reasonably linked to *Thermus*, which was a well-known As(III) oxidizer inhabiting geothermal environments (Fig. 6, Supplementary Fig. S2)^27^,^28^,^29^,^30^,^31^,^32^,^33^.

In addition to As, temperature, sulfide and DO were important environmental factors for determining the microbial community structure of the pools and downstream samples (Fig. 5). Due to the extremely high temperature, relatively low DO and high sulfide concentrations, the source waters from the pools were colonized by microaerophilic/anaerobic hyperthermophiles, such as *Thermocrinis*^34^, *Caldimicrobium*^35^, *Sulfophobococcus*^36^ and *Pyrobaculum*^37^ (Figs 5 and 7). The dominant *Thermocrinis* and *Caldimicrobium* in the pools are representative sulfur oxidizers, which are widely distributed in other alkaline sulfidic-rich hot springs around the world^15^,^38^. Along the outflow channel, where the temperature rapidly decreased, some microaerophilic thermophilic populations including *Ralstonia*, *Delftia* and *Undibacterium* of *Betaproteobacteria* were detected in high abundance in downstream samples with relatively high DO (Fig. 7 and Table 1)^39^. The shift from the sulfur oxidizer *Thermocrinis* in the source water to the thermophilic aerobic/microaerophilic *Persephonella*, *Hydrogenobacter* and *Thermus* downstream (Fig. 7), highlights the significant roles of temperature and DO in shaping the microbial community structure at this site^19^,^40^,^41^. In the sediment samples, *Proteobacteria* and *Crenarchaeota* dominated in the pools and some thermophilic photosynthetic bacteria, such as *Roseiflexus* and *Chloroflexus* of *Chloroflexi*, were predominant downstream (Figs 3 and 5), which is closer to their optimal growth temperature (<70 °C)^42^,^43^.

## Conclusions

High ratios of As(V)/As_Sum_ suggested As(III) oxidation occurred in the alkaline sulfide-rich Zimeiquan pool. As_Sum_ and total combined sulfide and sulfate concentrations substantially increased along the outflow channel, which implied the elevated As concentration was due to thioarsenic transformation. Temperature, sulfide, As and DO significantly influenced the microbial communities between not only the pools and downstream samples, but also water and sediment samples. In the upstream outflow channel, the dominant *Thermocrinis* transformed the thioarsenic to As(III), and then the downstream *Thermus* oxidized the derived As(III).

## Methods

### Site description

Zimeiquan (N24.95102°, E98.43613°), a representative alkaline sulfide-rich hot spring in the Rehai geothermal field of Tengchong, in Yunnan, southwestern China, was selected for this study (Supplementary Fig. S3). It has a pH of 9.0, sulfide concentrations up to 4.8 mg/L^22^, and As concentrations up to 655.6 μg/L^21^. Zimeiquan is comprised of two adjacent pools, with a depth of 4.5–9.5 cm and a length of 8.5–1.0 m. Spring water from the left pool flows into the right pool and then both are discharged along an outflow channel. Zimeiquan have a low water flow of 0.2–0.3 L/S. A transect of five sampling sites was established along the outlet. Sample identification was based on the relative distance in meters from the initial discharge point in the right pool (designated 0 m). The left pool was designated −1 m, and the downstream outlet samples were 4 m, 8 m and 12 m (Supplementary Fig. S3). Parallel water and sediment samples at each sampling site were collected in August, 2014.

### Field measurements and sample collection

Water temperature, pH and DO were measured in the field at the site of water collection using a hand-held meter. Concentrations of sulfide, ammonium, ferrous iron (Fe(II)) and total iron (Fe_Tot_) in the water were also determined in the field with a Hach spectrophotometer (DR850, Hach Corp., USA) according to the manufacturer’s instructions. Approximately 250 mL water samples were collected for laboratory measurements using disposable 50 mL syringes and filtered through a 0.22 μm polyethersulfone membrane syringe filters (to collect biomass) (Pall Corp., NY, USA) into 50 mL acid-washed polypropylene bottles (to measure anions and cations) and brown glass bottles (to measure thioarsenic and DOC). Water samples to be used for cations and DOC measurement were acidified with 1% v/v HNO_3_. The filters were placed into 15 mL sterile polypropylene tubes for later DNA extraction. Separation of As species was done *in situ*, following Le *et al*.^44^. Briefly, 10 mL of each water sample was applied to a silica-based strong anion-exchange cartridge (Supelco, USA) preconditioned with 50% methanol and deionized water before use. As(V) was retained in the cartridge and As(III) remained in the filtered solution. The As(V) was then eluted with 10 mL 1 mol HCl. Duplicate sediment samples were collected from each site using sterile spoons and transferred to sterile 50 mL polypropylene tubes that were immediately either stored on ice (for As-oxidizing microorganism isolation) or dry ice (for DNA extraction). All geochemical and DNA extraction samples were stored on dry ice in the field and during transportation, and then stored at −80 °C until analyzed.

### Laboratory geochemical analysis

Cation and anion concentrations were measured by inductively coupled plasma-optical emission spectrometry (CAP6300, Thermo, USA) and ion chromatography (ICS1100, Dionex, USA), respectively. Arsenic concentrations were determined using liquid chromatography-hydride generation-atomic fluorescence spectrometry (LC-HG-AFS, Haiguang AFS-9780, Beijing)^33^. Thioarsenic species were identified using Q Exactive, a high resolution quadrupole orbitrap mass spectrometer (Thermo Scientific, Germany), by detecting the accurate mass and matching the isotope abundance. DOC of water samples were determined using a TOC analyzer (TOC-V_CPH_, Shimadzu, Japan). As and Fe in the sediments were extracted by 1:1 aqua regia digestion in a water bath^45^. Extracted Fe from sediments was determined by a 1,10-Phenanthroline-based assay: 10 mL extracted solutions were mixed with 5 mL acetate-sodium acetate buffer (pH = 4.6), 2.5 mL 1% hydroxylamine hydrochloride and 5 mL 0.1% 1,10-phenanthroline solution in a 50 mL volumetric flask. The mixture was brought up to a volume of 50 mL with deionized water and allowed to stand for 10 min. The absorbance of each solution at 510 nm was measured with a spectrophotometer (UV1750, Shimadzu, Japan). TOC of sediment samples was measured with a Macro elemental analyzer (Multi EA 4000, Analytik Jena, Germany) after inorganic carbon was digested using HCl.

### As(III) oxidization experiments

Minimal salt medium (MSM) was used for enrichment of As(III)-oxidizer and assays for the detection of As oxidization under chemolithoautotrophic conditions. MSM contained (g/L): Na_2_SO_4_, 0.031; KH_2_PO_4_, 0.17; KCl, 0.15; MgCl_2_·6H_2_O, 0.04; CaCl_2_·2H_2_O, 0.05; (NH_4_)_2_SO_4_, 0.4; NaHCO_3_, 1.68; trace elements, 5 mL and vitamins solution, 10 mL^5^. The pH of the medium was adjusted to 8.5 to match that of Zimeiquan. About 2.5 mL of a water-sediment slurry at 8 m were inoculated in 50 mL MSM containing 3 mM NaAsO_2_. After one week incubation at 65 °C, ~25 mL of the culture was centrifuged for 5 min at 4000 rpm to collect biomass. The biomass was re-suspended in sterile water, and then inoculated in fresh MSM amended with 3 mM NaAsO_2_. An abiotic control was set up by adding sterile water to medium. Samples were taken periodically for As speciation. Experiments were run in triplicate.

### DNA extraction, amplification and sequencing

DNA was extracted from filters or from 0.5 g sediment samples or from enrichment product using the FastDNA SPIN Kit for Soil (MP Biomedical, OH, USA). DNA concentrations were measured by Pico Green using a FLUOstar OPTIMA fluorescence plate reader (BMG LABTECH, Jena, Germany). The V4 region of the 16S rRNA gene was amplified from DNA samples using the primer pair 515F (5′-GTGCCAGCMGCCGCGGTAA-3′) and 806R (5′-GGACTACHVGGGTWTCTAAT-3′) combined with Illumina adapter sequences, a pad and a linker of two bases, as well as barcodes on the reverse primers^46^. PCR amplification was carried out in a 25 μL reaction buffer containing 2.5 μL 10× PCR buffer II (including dNTPs) (Invitrogen, Grand Island, NY), 0.4 μmol of both forward and reverse primers, 10–15 ng DNA and 0.25 U high fidelity AccuPrime™ Taq DNA polymerase (Life Technologies) using the following program: initial denaturation at 94 °C for 1 min, followed by 30 cycles of 94 °C for 20 s, 53 °C for 25 s, and 68 °C for 45 s, and then a final extension at 68 °C for 10 min. Reactions were performed in triplicate and pooled. Positive PCR products were confirmed by agarose gel electrophoresis, then quantified with PicoGreen. Finally, 200 ng of PCR product from each sample were combined together and purified using a QIAquick Gel Extraction Kit (Qiagen, Valencia, CA) and then re-quantified with PicoGreen. Sample libraries for sequencing were prepared according to the MiSeqTM Reagent Kit Preparation Guide (Illumina, San Diego, CA, USA)^47^. Briefly, sample denaturation was performed by mixing 10 μL of combined PCR products (2 nmol) and 10 μL 0.2 mol NaOH and incubated for 8 min at room temperature. Denatured DNA was diluted to 15 pM using HT1 buffer and mixed with a PhiX DNA library (final concentration 14.3%). A total of 600 μL sample mixture, together with customized sequencing primers for forward, reverse, and index reads, were loaded into the corresponding wells on the reagent cartridge of a 500-cycle v2 MiSeq kit and run on an Illumina MiSeq system (Illumina, San Diego, CA).

The *aioA* genes were amplified from DNA extracted from the enrichments using degenerate primers aioA95f (TGYCABTWCTGCAIYGYIGG) and aioA599r (TCDGARTTGTASGCIGGICKRTT) in a 25 μL PCR mixture consisting of PCR Ex Taq buffer, 100 μmol dNTP mixtures, 0.5 μmol primers, 50 ng templates and 1 U of Ex Taq DNA polymerase (TaKaRa, Japan)^27^. The PCR program was as follows: initial denaturation at 94 °C for 5 min, followed by 9 cycles of denaturation at 94 °C for 45 s, annealing at 54 °C C for 45 s, extension at 72 °C for 1.5 min, and 25 cycles of denaturation at 94 °C for 45 s, annealing at 50 °C for 45 s, extension at 72 °C for 1.5 min, and then a final extension step at 72 °C for 7 min^27^. PCR product was purified using the E.Z.N.A. Gel Extraction Kit (Omega Bio-tek, Inc. GA, USA). The purified PCR product was ligated into pMD-18T vectors (TaKaRa) and transformed into *Escherichia coli* DH5a competent cells. The transformed cells were plated on Luria-Bertani (LB) plates containing 100 μg/mL of ampicillin, 80 μg/mL of 5-bromo-4-chloro-3-indolyl-b-D-galactopyranoside (X-Gal) and 0.5 mmol isopropyl-b-D-thiogalactopyranoside (IPTG), and incubated overnight at 37 °C. Twenty-six randomly chosen white colonies were sequenced with an ABI 3730 automated sequencer.

### Sequence data preprocessing and statistical analysis

Raw 16S rRNA sequences with perfect matches to barcodes were split to sample libraries and were trimmed using Btrim with a QC threshold of greater than 25 over a 5 bp window size and a minimum length of 150 bp^48^. Forward and reverse reads with at least 50 bp overlap and less than 5% mismatches were joined using Fast Length Adjustment of SHort reads (FLASH)^49^. After trimming of ambiguous bases (i.e. N), joined sequences with lengths between 247 and 258 bp were subjected to chimera removal by Uchime^50^. Clustering of operational taxonomic units (OTUs) was performed by Uclust at a similarity level of 97%^51^, and taxonomic assignment was through the Ribosomal Database Project (RDP) classifier with a minimal 50% confidence estimate^52^. Samples were rarefied at 15 000 sequences per sample. Singletons in generated OTU tables were removed for downstream analyses. The above steps were performed through a Galaxy-based pipeline at the Institute for Environmental Genomics, University of Oklahoma (http://zhoulab5.rccc.ou.edu/). All statistical analyses in this study were performed based on genus-level OTUs at a 97% similarity level with the Vegan package in R (http://www.r-project.org/), unless otherwise stated. A variety of alpha diversity indices were calculated including Chao1, Shannon and Equitability. Hierarchical clustering trees using the unweighted pair group method with arithmetic means (UPGMA), principal coordinates analysis (PCoA) and non-metric dimensional scaling (NMDS) ordination plots were built to depict the community structure based on the the Bray-Curtis dissimilarity matrix of detected OTUs. The Envfit function in the Vegan package was used to overlay significant environmental variables on the NMDS ordination. Analyses of similarity (ANOSIM), non-parametric multivariate ANOVA (ADONIS) and multi-response permutation procedure (MRPP) were performed to test for significant differences of microbial community composition between sample types (i.e., water vs. sediment) and different locations (i.e., springs vs. downstream). DNA sequences were deposited to the Short Read Archive database at NCBI (Accession number: SRP059839).

The *aioA* gene nucleotide sequences were edited in MEGA 5.05 and binned into various operational taxonomic units (OTU) using DOTUR 1.53. A representative sequence from the one OTU (0.01 cutoff) identified was selected for phylogenetic analysis. Prior to phylogenetic analysis, the representative *aioA* gene sequence was translated into amino acid sequences and compared with closely related AioA amino acid sequences in the GenBank database (BLASTX), which were then used to construct a phylogenetic tree with MEGA 5.05. The *aioA* gene fragment nucleotide sequences were deposited in the GenBank database under accession number KU311042.

## Additional Information

**How to cite this article**: Jiang, Z. *et al*. Microbial communities and arsenic biogeochemistry at the outflow of an alkaline sulfide-rich hot spring. *Sci. Rep*. **6**, 25262; doi: 10.1038/srep25262 (2016).

## Supplementary Material

Supplementary Information

## Figures and Tables

**Table 1:** Geochemistry of parallel water and sediment samples collected along the Zimeiquan outflow channel in Tengchong geothermal area.

Distance from discharge (m)	Aqueous phase													Solid phase		
			mg/L		μmol/L									mg/kg		
	T °C	pH	DO	DOC	Ammonia	Nitrate	Sulfide	Sulfate	Fe_Tot_	As(III)	As(V)	As_Sum_^a^	As(V)/As_Sum_	Fe_Tot_	As_Tot_^b^	TOC(%)
−1	93.6	8.09	0.36	3.43	9.44	bdl	156.25	236.14	bdl	3.45	4.88	8.33	0.59	543.90	87.97	0.28
0	90.3	8.12	1.04	1.75	10.00	bdl	183.44	245.18	bdl	1.20	4.25	5.45	0.78	970.75	72.35	0.17
4	68.6	8.46	0.61	2.01	3.33	bdl	35.94	256.08	bdl	9.89	3.84	13.73	0.28	29.39	84.37	2.02
8	64.7	8.51	2.79	2.02	1.11	6.51	2.81	326.12	bdl	7.71	6.16	13.87	0.44	278.06	22.15	1.21
12	49.6	8.87	3.30	0.80	bdl	bdl	0.31	363.97	bdl	7.26	6.59	13.86	0.48	268.40	67.51	2.60

^a^As_Sum_ refers to the sum of As(III) and As(V).

^b^As_Tot_ refers to total As concentration extracted from sediments. bdl: below detection limit (1 μg/L).
