# Supplementary material for: Microbial communities and arsenic biogeochemistry at the outflow of an alkaline sulfide-rich hot spring
Source: Sci Rep. 2016 Apr 29;6:25262. doi: 10.1038/srep25262 (PMC4850476; doi:10.1038/srep25262)
Supplement: Supplementary Information [file srep25262-s1.pdf]

Microbial communities and arsenic biogeochemistry at the outflow of an alkaline sulfide-rich hot spring

Zhou Jiang[1,2], Ping Li[1,*], Joy D. Van Nostrand[3], Ping Zhang[3], Jizhong Zhou[3,4,5], Yanhong Wang[1], Xinyue Dai[1,2], Rui Zhang[1], Dawei Jiang[1], Yanxin Wang [1,2,*]

[1] State Key Laboratory of Biogeology and Environmental Geology, China University of Geosciences, Wuhan, 430074, China

[2] School of Environmental Studies, China University of Geosciences, Wuhan, 430074, China

[3] Institute for Environmental Genomics and Department of Microbiology and Plant Biology, University of Oklahoma, Norman, OK, 73019, USA

[4] Earth Sciences Division, Lawrence Berkeley National Laboratory, Berkeley, CA, 94720, USA

[5] School of Environment, Tsinghua University, Beijing, 100084, China

* Corresponding authors: pli@cug.edu.cn, yx.wang@cug.edu.cn

Tel: +86-27-67883452; Fax: +86-27-67883456

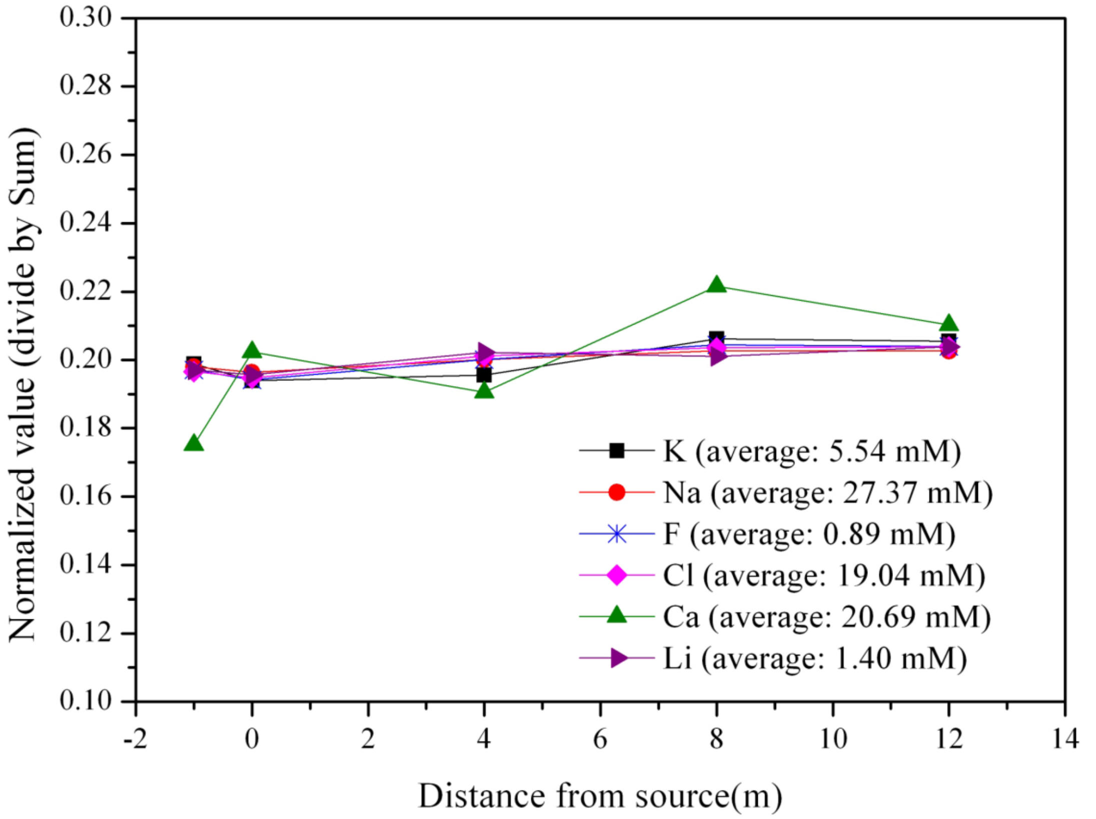

Supplementary Figure S1Normalized main cations and anions concentrations (divided by the sum) variation along the outflow channel of Zimeiquan. The values in parenthesis were averaged concentrations of ions with units of μM and mM.

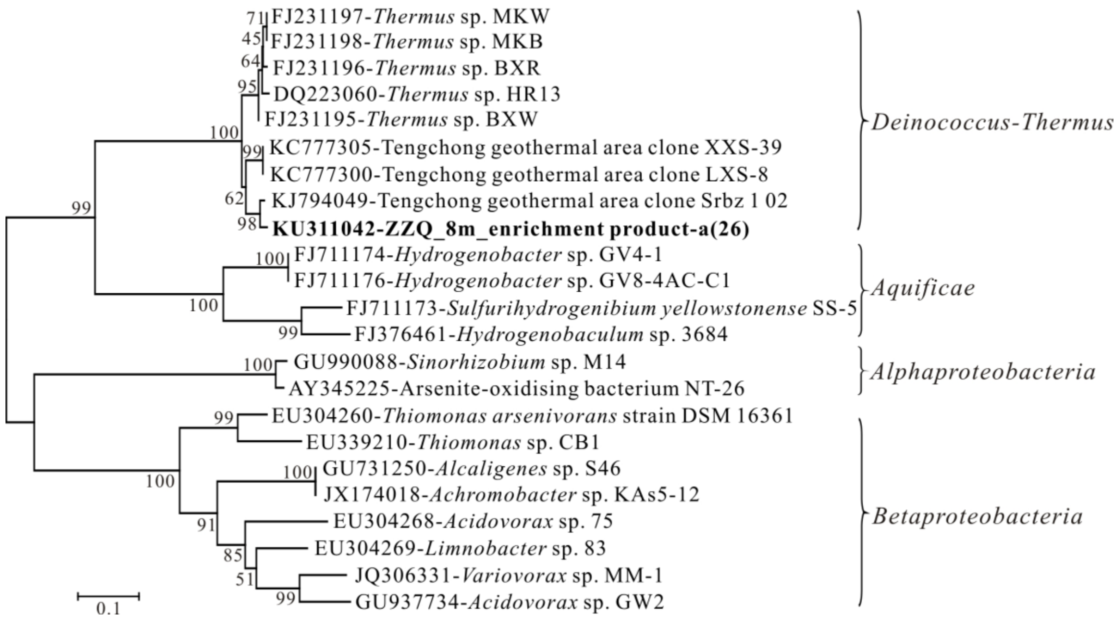

Supplementary Figure S2 Phylogeny of *aioA* sequences (~165 unambiguous amino acids) deduced from enrichment product of Zimeiquan downstream samples at 8 m. One representative type within each phylotype was shown. Lower-case letters showed the variety of OTU. Numbers in parentheses indicates the number of sequences obtained for each sequence type.

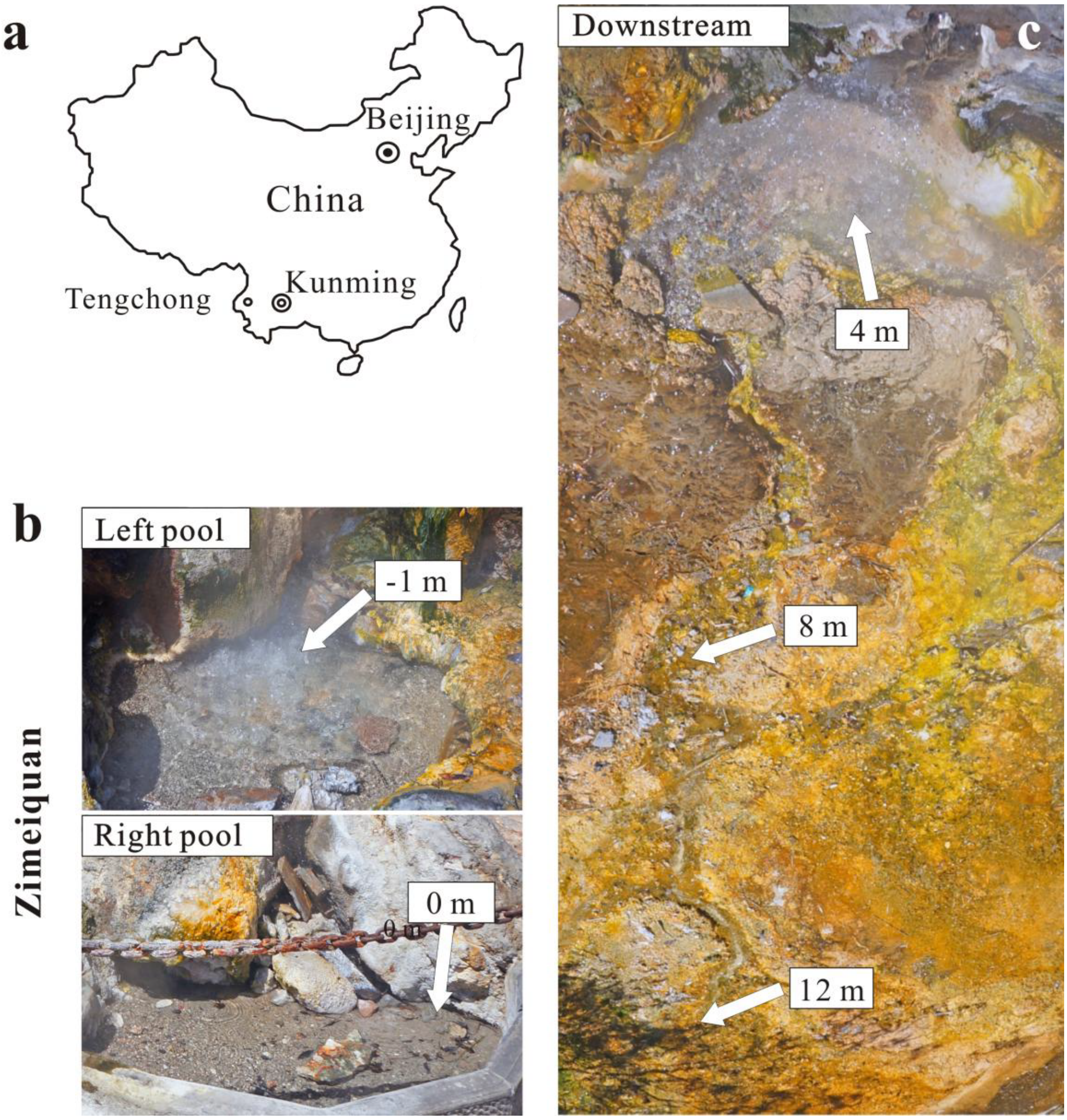

Supplementary Figure S3 A map showing the location of Tengchong in China (a), Zimeiquan sampling sites located in Rehai, Tengchong geothermal area (b) and its downstream sampling sites (c). Figure S2a was depicted in the background of China map by using CorelDRAW® Graphics Suite X7 (http://www.coreldraw.com/cn/)

Supplementary Table S1 Alpha diversity indices at a 97% similarity level of 16S rRNA gene fragments by re-sampling 15000 reads in each sample

Samples	Distances	Observed OTUs	Coverage[a] of observed OTUs (%)	Chao1	Shannon' diversity	Equitability
Water	-1 m	277	66.98	414	2.23	0.40
	0 m	569	84.74	672	3.42	0.54
	4 m	754	64.63	1167	3.42	0.52
	8 m	395	76.52	516	3.21	0.54
	12 m	539	68.62	786	3.37	0.54
Sediment	-1 m	334	78.58	425	3.38	0.58
	0 m	380	76.15	499	3.48	0.59
	4 m	457	72.03	634	3.45	0.56
	8 m	380	88.99	427	2.90	0.49
	12 m	547	89.96	608	4.36	0.69

[a] Coverage is the ratio of the observed OTUs to Chao1

Supplementary Table S2 Significance tests of microbial community structure difference between different groups with three complementary statistical approaches.

Data sets	adonis[a]		ANOSIM[b]		MRPP[c]	
	F	*P*	*R*	*P*	δ	*P*
Water vs. Sediment	2.1112	0.026	0.1721	0.149	0.7766	0.016
Pools vs. Downstream[§]	2.6661	0.021	0.4246	0.032	0.7486	0.012

All tests are non-parametric multivariate analyses based on Bray-Curtis dissimilarities among samples. [§] Pools: samples from -1 m and 0 m, downstream: samples from 4 m, 8 m and 12 m. [a] adonis: Permutational multivariate analysis of variance using distance matrices. Significance tests were carried out using F-tests based on sequential sums of squares from permutations of the raw data. [b] ASNOSIM: Analysis of similarities which provides a way to test statistically whether there is a significant difference between two or more groups of sampling units. Statistic R is based on the difference of mean ranks between groups and within groups. The significance of observed R is assessed by permuting the grouping vector to obtain the empirical distribution of R under the null model. [c] MRPP: Multi-response permutation procedure. Statistic delta is the overall weighted mean of within-group means of the pairwise dissimilarities among sampling units. The significance test is the fraction of permuted delta that is less than the observed delta. More than two complementary approaches with $p < 0.05$ displays the significant difference of microbial community structures between above groups.
